# The Duty of Texas Legislators on Sanitation

**Published:** 1891-02

**Authors:** O. Eastland

**Affiliations:** Wichita Falls


For Daniel's Texas Medical Journal.

### THE DUTY OF TEXAS LEGISLATORS ON SANITATION.

BY O. EASTLAND, M. D., WICHITA FALLS.

SOME ONE has said that the medical profession of Texas expects much of the present legislature now in session at the capital; joined to which sentiment I wish to say this: they will not disappoint us if they can see the matter in its true light.

Sanitation in its broadest sense encompasses the regulation of the practice of medicine, for the competence or incompetence of those who are in charge of the health of the inhabitants of the State is a constant guard, or on the other hand, a menace to its welfare.

Previous legislatures have passed the subject lightly by, and to-day the statutes of the grandest, and in other things the most progressive State in the Union, stands a practical blank in this direction, so that whoever desires to assume the title, doctor, may do so with a full degree of assurance that none may molest; the ordinary citizen having nothing save the name and appearance, often deceptive, to guide him in the choice of medical counsel. Sanitation, in its more specific application, concerns itself with those preventable diseases which are constantly interfering with the physiological state of the human organism.

Now, with these two aspects of the subject before us, let thought turn upon the mode of adjusting means to ends. Why in the past has this important subject been so neglected by our senators and representatives? The answer to this query, when put to the minds of all members of the medical fraternity who have given it serious thought, must be in unison and briefly, is this: Our senators and representatives have not appreciated its importance; they are not informed on the true inwardness of the subject. But you say they are intelligent men, jurists, statesmen, financiers, etc., as good as any State under the flag can boast; and all this I grant most freely and with due pride; but the barrier to a proper conception of the subject's importance and recognition is the financial point. Finance is the word which solves the problem, as my intercourse with legislators has proven. They are men who, with commendable zeal seek to save the public treasury of Texas every dollar they can, and when you say, "Board of Health," "Texas College of Physicians and Surgeons," and other suggestions of advanced medicine, they say at once, "I am interested in the matter you propose, but what does it cost?" Now, the duty of the medical profession to the Texas legislator is upon them; here is your opportunity to lay before them, upon a basis of dollars and cents, the advantages to the State, of such legislation, leaving entirely out of the question the humanitarian side.

We have indisputable statistics from States now having in operation just such sanitary regulations, etc., as we propose, and such States find it a good investment on the basis of the relative value to the State of a sick man and a well man. The statistics of these States show a certain number who were affected each year from certain preventable diseases before the enactment of sanitary laws, and a given number since; the difference being great; insomuch that the financiers who calculate the lost time of a sick man worth so much to the aggregate production of wealth then the time of others consumed in nursing and caring for the sick one, and they find the cost of maintaining such laws is more than repaid into their several State treasuries each year.

Is there any doubt in the mind of any member of the State profession as to the results of such a conception of the problem before us by our legislators?

A people must be educated; a nation's characteristics are formed by training and influence, and the several professions and various vocations are the sources looked to for education in each particular way, and it is ours to elucidate the needs of Texas in a sanitary way; make it plain on a basis of dollars, and then place on this the superincumbent weight of the humanitarian aspect of it, and the results will follow, when the minds of our legislators are so impressed by us.

Having laid down these facts, which are incontrovertible, can not each do a part toward giving them lodgment in the minds of our legislators, whose proper conception of the question will bear the fruit desired?

Having done with these thoughts, I hope to be pardoned for attempting a forecast of national medical laws, and government in medical research, in which, as a nation, we are as far behind some of the European powers as Texas is behind some of her sister States in State sanitation. My last summer's travel and study in Europe served to extract a good measure of national egotism from me, for, like most Americans, I boarded the trans-Atlantic steamer filled with the idea that we had at home the best of everything, except, possibly, our institutions of learning had not yet attained, by age, that plane of excellence which can only be gained by the mental toil and discipline of centuries. But when I came face to face with the results of Koch's researches at Berlin, under the direction of the German government, which has not been sparing in funds to enable him to devote the entire force of his well trained intellect in this channel, and it was a knowledge of this fact that electrified the thousands of listening ears at the International Medical Congress when he hinted at what has since been brought forward as one of the foremost discoveries of the age.

Another notable example, under wise provisions of the French government is found in Paris, where thousands of francs have been spent acquiring valuable ground in the heart of the city on which has been built, at a cost of many more thousands of francs the Pasteur Institute for the treatment of hydrophobia. Its expensive laboratories and other complete equipments, stand as an enduring monument of the wisdom of the legislators of France, and Pasteur, in his ripe old age, sees from one hundred to one hundred and fifty daily, from all parts of Europe, come for the anti-hydrophobic inoculation which has reduced the death rate so decidedly.

It was when I had seen these things that my egotism vanished, and in this line of a progressive era the "stars and stripes" floated far back in the procession. In my chagrin I questioned: Why? The correct answer to which seems to be that our congressmen are not educated in the matter; but all absorbing questions, such as high and low tariff, silver coinage, election laws, etc., by the dozens; but it is only now and then when yellow fever on our southern coasts puts out its devastating hand, that they find any thought for the real foundation of national prosperity—the health of its inhabitants. There are other diseases quietly in progress all over these United States, which in the aggregate, destroy more life than these virulent epidemics, diseases which might be placed in check if only their natures were properly understood; but it cannot be expected of any individual member of the profession that he can lay aside, at a personal sacrifice, financially, his daily duties to study and investigate some germ of disease, its habitat, environments under which it multiplies most rapidly for interference with the health of the inhabitants of the land. But I shall see our congress follow the examples noted on the other continent, if only we shall demonstrate to its component elements the real value to the country of the expenditure of sums of money, however large, to enable careful and conscientious investigators to devote their entire cerebral force to one thing until it shall have been understood in its minutest detail.

The attainment of such an end is worthy of the most assiduous efforts of every member of the profession in the broad expanse of our Union.

